# A Diary Study on When and With Whom Recovery Experiences Modulate Daily Stress and Worry During a COVID-19 Lockdown

**DOI:** 10.3389/fpsyg.2021.620349

**Published:** 2021-04-16

**Authors:** Julie Ménard, Annie Foucreault, Hugues Leduc, Sophie Meunier, Sarah-Geneviève Trépanier

**Affiliations:** ^1^Department of Psychology, Université du Québec à Montréal, Montréal, QC, Canada; ^2^Department of Management, Université du Québec à Trois-Rivières, Trois-Rivières, QC, Canada

**Keywords:** coronavirus outbreak, COVID-19, self-isolation, mental health, pandemic, mood, lockdown

## Abstract

In April 2020, almost six out of 10 people around the world were in lockdown due to the COVID-19 pandemic. Being locked down usually has a deleterious effect on the confined individual's mental health. In this exceptionally challenging context, finding ways to minimize negative mood about the pandemic is essential. Pandemic-related negative states (“negative mood”) and recovery experiences were investigated in a sample of 264 individuals who completed daily surveys four times per day over 7 consecutive days. MSEMs analyses revealed that negative mood persisted from moment-to-moment through the day, thus showing a response lag effect. Further analyses revealed that when someone experienced pandemic-related psychological detachment, relaxation, mastery, control, pleasure, or relatedness at specific periods of the day, mood had improved at the next measured time period, suggesting a protective effect. However, the pattern displayed by singles with dependents suggests that some recovery experiences at specific periods during the day seem to have a backfiring effect and worsen subsequent mood. These findings bring new insight into the role of recovery experiences during lockdowns and suggest that many could benefit from such experiences throughout the day when self-isolating. However, for individuals with multiple risk factors such as being single with dependents, some recovery experiences, at specific periods during the day, might not bring the desired outcome and future research is needed to examine if guilt or domestic burden may explain this finding. Results contribute to our understanding of how to take care of one's mental health during the current pandemic, and concrete recommendations adapted to individual contexts are provided.

## Introduction

Although shutdown of non-essential services and voluntary self-isolation are effective means for protecting the physical health of individuals during the COVID-19 pandemic, their consequences can be detrimental to mental health (Gao et al., 2020; Holmes et al., 2020; Pietrabissa and Simpson, 2020; Qiu et al., 2020; Rubin and Wessely, 2020; Yu-Tao et al., 2020). From 1 day to the next, many workers have thus been forced to work from home [35.2% in the US workforce (Akkermans et al., 2020)]. Others have faced immediate job loss or greatly reduced work hours (Akkermans et al., 2020), which means they have less income or none at all, as well as being affected by a large-scale economic downturn (Nicola et al., 2020). Furthermore, closing workplaces, schools, daycares, and institutions of higher learning had major consequences on the daily organization of family life. The entire usual routine has been disrupted (Brooks et al., 2020). According to the United Nations Educational, Scientific and Cultural Organization, approximately 1.38 billion children were out of school or childcare (Cluver et al., 2020). The daily life of parents of preschool and school-age children has changed notably, since they had to look after their children 24 hours a day, 7 days a week with no outside support, since that would have contravened physical distancing norms; the same is still true for group activities, team sports, and playgrounds. Some of these parents are expected to work remotely, while others are unable to work, without knowing how long the situation will last. In this context, just keeping children busy and safe is a constant overwhelming prospect, especially for those who have lost their jobs, have a low income (Cluver et al., 2020) or have little social support, such as single parents (Wang et al., 2020; Zhou et al., 2020). In addition, going out and meeting with others are restricted by lockdown measures and physical distancing, and can generate stress and worry due to a feeling of loss of freedom and isolation from the rest of the world (Brooks et al., 2020). Thus, most residents of countries affected by this pandemic had to make major changes to all spheres of their lives, in addition to being constantly reminded that a potentially deadly virus is present and spreading, which can lead to stress and worry (Park et al., 2020). This can have important consequences for the population's health, especially since stress and worry are states that persist over time if nothing is done to counteract them (Thoits, 2010). In fact, we already know that stress and worry tend to have a response lag effect over time in the same person, from 1 day to the next (Affleck et al., 1994; van Eck et al., 1998). Researchers have also found that on a daily basis, for some people, mood tend to persist from one moment to the other, a phenomenon that have been labeled as emotional inertia (Suls et al., 1998; Kuppens et al., 2010). In contrast, we know very little about the response lag effect from one time of day to another, in the context of a pandemic. Since these extraordinary circumstances require constant adaptation and add pressure and uncertainty to daily life, we can assume that a person who feels stress or worry tends to remain in this state from one period to the next over the course of the day, and will have difficulty recovering from daily strain. This must be tested empirically, however.

Therefore, the primary purpose of this study is to determine if there is a moment-to-moment response lag effect from one period of the day to the next [i.e., the lagged effect of prior mood on subsequent mood in which prior mood leaves a residue (Marco and Suls, 1993)] of individuals' experience of pandemic-related negative mood (i.e., stress and worry) over the course of the same day among those who are in voluntary isolation. The secondary purpose is to identify proven recovery strategies for lowering the strain experienced by individuals in this situation (i.e., psychological detachment, mastery, control, relaxation, pleasure, and relatedness) that would attenuate such a response lag effect and thus contribute to promoting daily well-being among people in self-isolation. The third purpose is to determine when (i.e., in the morning, in the afternoon, in the evening, at bedtime) and for whom these strategies are more effective.

*Hypothesis 1*. Prior momentary pandemic-related (a) stress and (b) worry will have a lagged effect on the next time period, throughout the day. There will be a positive relationship between prior mood and mood at the next time period.

If such a response lag effect from one moment to another during the day exists, it is essential and urgent that effective means for reducing states of stress and worry be identified, in order to foster the recovery of persons who must constantly adapt to a new reality. To be useful, these must be simple measures, accessible to everyone and applicable in a context of physical distancing, so as to preserve the physical and mental health of all those affected by self-isolation during the COVID-19 pandemic. Investigating recovery experiences seems particularly appropriate in this regard.

The Conservation of Resources theory (COR; Hobfoll, 1989) provides valuable insight into the process underlying recovery from strain. According to this perspective, individuals seek to obtain, preserve, build and protect their personal resources, which are essential to their psychological health. According to COR, stress is the result of the loss (real or anticipated) of resources invested to adapt. The more resources a person possesses, the less they are likely to find themselves in a deficit situation (stressed or worried). Conversely, the fewer resources a person has available to them, the more they are at risk, since using resources to adapt is riskier for a person who has little. If using resources causes significant loss, the person can find themselves in a spiral of loss, since they will not have sufficient resources to invest to “get out of a hole.” To preserve mental health, too great a loss of resources must be avoided and any lost resources must be restored during a process of adaptation. Recovery experiences can do just that, allowing the recovery of personal resources lost during daily life (Sonnentag et al., 2008). Recovery experiences play a functional role in resource recovery that is more relevant than specific activities since individuals differ with respect to the activities they experience as recovering but not in regard to the underlying psychological process of recovering (Sonnentag and Fritz, 2007). For example, taking a bath may not be as restorative as watching a series for someone whereas someone else may have the opposite experience.

A total of six different recovery experiences were identified in the literature and examined in this study (Siltaloppi et al., 2009; Fritz et al., 2010; Shimazu et al., 2012; de Bloom et al., 2015; van Hooff and de Pater, 2017; Bosch et al., 2018). Studies indicate that these recovery experiences are related to fewer negative moods, such as psychological distress and exhaustion, and enhanced well-being and positive mood (van Wijhe et al., 2013; Bosch et al., 2018; Sianoja et al., 2018; Horan et al., 2020). First, psychological detachment refers to mentally distancing oneself from anything that may remind one of the activity that provokes strain [e.g., work (Sonnentag and Fritz, 2015)]. Concretely, in the context of the pandemic, detachment means ceasing to be exposed to pandemic-related issues (e.g., daily press conferences, having a conversation about COVID-19, etc.) and no longer thinking about the virus and its consequences. In this regard, Liu (2020) showed that seeking out and absorbing information about COVID-19 through digital media increases worry. Ceasing to actively search for and pay attention to information about the pandemic could thus potentially make it possible to avoid becoming more worried about the current context and enable a certain degree of psychological detachment. Second, relaxation involves doing something that generates positive emotions with a low investment of effort (Sonnentag and Fritz, 2007). Relaxation can foster feeling good despite the pandemic, since expending little energy allows the body and mind to become re-energized and replenished with new resources (e.g., vigor, concentration, and the ability to cope with challenges). Third, mastery experiences occur when a person does something to broaden their horizons (Sonnentag and Fritz, 2007). By providing people with opportunities to learn or challenge themselves, these activities can enable them to grow despite the pandemic. For example, having new experiences such as making bread or learning about a subject of interest (other than COVID-19) could also help minimize pandemic-related negative mood. Fourth, experiences of control involve determining for oneself how to occupy one's time. These experiences are beneficial because they enable making choices based on our preferences and needs (Sonnentag, 2018). Thus, to be able to decide when and how to engage in different activities over the course of the day could have a positive effect on mental health during self-isolation. Fifth, pleasurable experiences occur when a person experiences positive emotion such as joy, happiness and fun. Research shows that these emotions are beneficial because they stimulate the release of hormones (i.e., serotonin and dopamine) that reduce stress reactions (Esch and Stefano, 2004; Hooff et al., 2011). Activities that give one pleasure, such as engaging in one's favorite hobby, may thus have the potential to decrease negative mood (Sianoja et al., 2018). Finally, relatedness experiences refer to feeling close and connected to others. They involve the perception of belonging to a group in which the person feels comfortable sharing both their joys and worries (Van den Broeck et al., 2008). Thus, having the opportunity to chat with significant others during self-isolation can have a positive effect on mood. In summary, each of these six experiences has the potential to help people feel good despite the lockdown, as they provide opportunities to build and replenish resources that contribute to their well-being (e.g., love, self-esteem, advice, etc.).

*Hypothesis 2*. Recovery experiences will moderate the within-person lag effect of pandemic-related stress between time periods. The positive relation between stress during the previous time period and subsequent stress will be weaker when psychological detachment (H2a), relaxation (H2b), mastery (H2c), control (H2d), pleasure (H2e), and relatedness (H2f) are high (vs. low).

*Hypothesis 3*. Recovery experiences will moderate the within-person lag effect of pandemic-related worry between time periods. The positive relation between worry during the previous time period and subsequent worry will be weaker when psychological detachment (H2a), relaxation (H2b), mastery (H2c), control (H2d), pleasure (H2e), and relatedness (H2f) are high (vs. low).

Finally, it is crucial to evaluate the specific context of single persons with dependents. According to the work-home resources model (W-HR, Ten Brummelhuis and Bakker, 2012), single persons and those with dependents may report the highest levels of psychological distress due to the scarcity of social support they received to respond to family-related demands compared to those in a relationship. In fact, social support has been identified as a key contextual and volatile resource that can generate a gain spiral (Ten Brummelhuis and Bakker, 2012). A gain spiral is a phenomenon in which resources, such as social support, generate other resources, such as positive mood and enhanced self-esteem (Hobfoll, 1989; Ten Brummelhuis and Bakker, 2012). Since voluntary self-isolation has limited the contact parents can have with people outside their family unit, marital status should be investigated as it was the only potential source of support for many parents with dependents. We thus conducted an exploratory study comparing these two groups, to investigate whether marital status (single, vs. in a relationship) and the fact of having dependents or not would modify the effects of recovery experiences on lag effect of negative mood.

## Materials and Methods

### Procedure

This study was approved by the Institutional Review Board of the researchers' institution. Participants were volunteers and provided written consent to participate in this study. They were recruited through posts on personal (e.g., Facebook) and professional (e.g., LinkedIn) social media discussion groups on topics related to COVID-19. Members of these networks were invited to circulate the post among their contacts. To preserve participants' anonymity, those interested were invited to click on a link in the post to the platform SurveyMonkey. The initial questionnaire had to be completed between April 9 and 13, 2020. In this questionnaire, participants were asked to answer socio-demographic questions and to indicate the email address to which the diaries should be sent.

From April 14 to 20, 2020 (Tuesday morning to Monday evening), participants had to complete online diaries four times a day (between 5 a.m. and 2:30 p.m.; 12 and 6:30 p.m.; 4 and 10:30 p.m.; 8 p.m. and 7:30 a.m.). As de Vries et al. (2001) highlighted, time-based studies may uncover diurnal variations in the patterns of stress and the onset of worry. We could not and did not aim to impose strict times for completing the diaries, since schedules during lockdown tend to be more flexible and vary more widely from one person to another (Aymerich-Franch, 2020); e.g., fewer individuals have fixed schedules based on their work or school routines or their children's schooling). It is important to note that participants came from different provinces within Canada, with different time zones. This is why we had a 2.5 h overlap between the different measurement times. However, we did limit the overlap between measurement times to a maximum of 2.5 h by automatically closing the online questionnaires when the time span was elapsed. Prospective daily life studies such as this one have the advantage of assessing time and contextual effects and their ecological validity, while minimizing retrospective distortions and taking into account within and between-person variability (de Vries et al., 2001). This study's design covered 7 consecutive days and 28 measurement times, providing a relevant sample of a person's everyday life over a full week in lockdown. Indeed, it is ecologically valid because it was carried out in their natural environment and involved situations and activities from daily life during voluntary self-isolation (de Vries et al., 2001). “If it is our goal to provide true-to-life descriptions of stress and anxiety, research has to be carried out in the natural environment or in carefully selected settings, having similar connotations as daily life situations” (de Vries et al., 2001, p. 290), and this is what we did.

Current mood (i.e., pandemic-related stress and worry), and recovery experiences were assessed in these online diaries. Because recollections of experiences increasingly reflect stable attributes of the person over time rather than actual experience (Robinson and Clore, 2002), participants were required to report their immediate state (i.e., current pandemic-related stress and worry) and their experience in the very recent past (i.e., recovery experiences during the past few hours).

### Participants

In total, 423 individuals volunteered and filled out the initial questionnaire. To be included in the final study sample, participants had to live in Canada and be in voluntary self-isolation because of the COVID-19 pandemic (i.e., avoid leaving your home except for the purchase of essential goods or for outdoor outings while keeping a safe distance). Individuals were excluded from the study sample if they were working outside the home, in order to ensure a more homogenous sample in regard to potential sources of stress and worry [e.g., this excluded persons working in the health care sector, who are exposed to a high level of health risk (ILO, 2020)]. Individuals were also excluded if they were in lockdown for a reason other than the pandemic (e.g., incarceration). Based on these exclusion criteria, 87 persons were removed from the sample. As this study examines the lagged effect of prior mood on subsequent mood throughout specific periods of the day, only individuals for whom each intra-day transition was observed at least once were retained in the study sample (i.e., 72 individuals were removed based on this criteria). The final study sample comprised 264 participants aged 21–81 years (*M* = 42.35; *SD* = 13.36). On average, the response rate per measurement time (average of 77%; *M* = 205; *SD* = 20.47; min = 165; max = 245) was satisfactory (Babbie, 1990). Little's MCAR test indicates that the data missing at each measurement times were entirely random: χ^2^ = 34139.93 (*df* =36151, *p* = 1.000). Among participants, 86.4% were women and 59.5% were married or with a partner. The majority had a University diploma (82.2%) and was still employed at the time the study was conducted (29.9% were working from home full-time, 16.7% part-time, and 6.4% were self-employed and working from home). In total, 37.1% of participants had at least one dependent from 0 to 96 years old *(M* = 17.40; *SD* = 22.79). They worked in various sectors, including educational services (17.8% of participants), professional, scientific and technical services (9.8%), and health care and social services (8%).

### Self-Report Questionnaires

Given the importance of retaining participants over time while minimizing their level of burden and stress during the pandemic, all scales were abridged following the recommendations of Coste et al. (1997). More precisely, based on the results of the validation studies of the original scales, we used an expert-based approach (i.e., most representative item, conceptual consistency of the item, face validity of the item in a pandemic context) combined with a statistical approach (i.e., items with higher factor loading, less cross loading and higher contribution to internal consistency of the scale) to select one item per scale.

### Pandemic-Related Momentary Negative Mood

At each measurement time, participants had to indicate their current level of pandemic-related negative mood on a Likert scale from 1 (*not at all*) to 5 (*very much*). Items were created to meet the needs and goals of this study (2 items; “Now, I am stressed about the pandemic,” “Now, I am worried about the pandemic”; average data points: 5,762).

### Recovery Experiences

To assess recovery experiences over the last time period, we selected 1 item per subscale from the Recovery Experience Questionnaire (Sonnentag and Fritz, 2007). Items are: “I distanced myself from the pandemic” (pandemic-related psychological detachment), “I did relaxing things” (relaxation), “I did things to broaden my horizons” (mastery), “I determined for myself how I spent my time” (control). Social relatedness was assessed using one item adapted from the scale developed by van Hooff et al. (2018); “I did things that made me feel close and connected to others.” The item for pleasure was created to achieve this study's aim and follows the structure of the Recovery Experience Questionnaire (i.e., “I did things that made me happy”). Participants were asked to indicate to what degree each of the items corresponded to their experience over the last time period of the day, on a scale of 1 (*not at all*) to 5 (*very much*), three times per day (these items were not included in the first diary; average data points = 4,261).

### Contextual Variables

We assessed marital status (0 = single, separated, divorced, or widowed; 1 = married or in a relationship) and the extent to which participants took care of dependents over the last time period [item: “This (last time period), I had to take care of a dependent (e.g., child, someone who is sick or physically impaired; 0 = no; 1= yes) as these two variables have been shown to have an influence on mental health during the pandemic (Wang et al., 2020)”].

### Statistical Analyses

**Figure 1:**
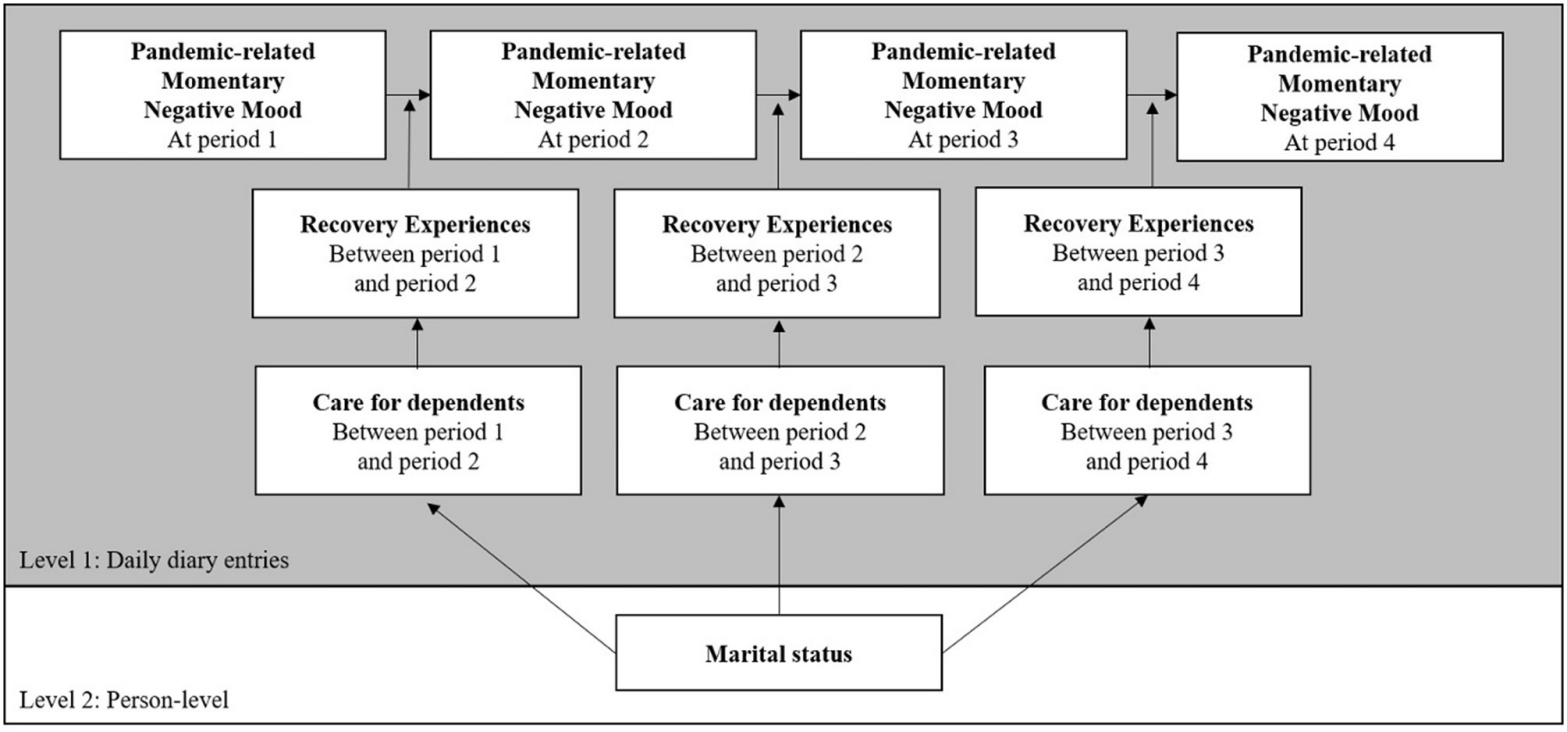
The conceptual model of within-person reciprocal relationships among pandemic-related momentary negative mood and recovery experiences.

We specified multi-level structural equation models (MSEM) using Mplus 8 (Muthén and Muthén, 2017) software to take into account the nested data structure, with daily diary entries (Level 1) nested within persons (Level 2). Combining structural equation modeling with analysis of hierarchical data, MSEM enables variables and their effects to be decomposed into between- and within-person components (Muthen and Satorra, 1995). Our hypotheses were tested via a two-level model with fixed slopes at Level 1 (one for each combination of mood and recovery strategies). Since we were interested in within-person relations, day-level predictor and control variables (Level 1; stress, worry, recovery experiences and taking care of a dependent) were centered around the person mean (group mean centering). At Level 2, we added marital status as control variable, centered around the grand mean prior to analysis (Enders and Tofighi, 2007). The paths shown in the theoretical model depicted in Figure 1 were modelized.

## Results

### Preliminary Analyses

Means, standard deviations, correlations, and intraclass coefficients (ICCs) are presented in Table 1. ICCs ranged from 0.29 to 0.72, confirming the appropriateness of MSEM (see Verbeke and Molenberghs, 2000, p. 25). Also, correlations indicated that almost all daily recovery activities were negatively associated with daily stress and daily worry. The only exceptions were the non-significant association between daily mastery and daily stress (levels 1 and 2) and worry (level 2) and between daily relatedness and daily worry (level 2).

**Table 1:** Descriptive statistics, correlations, and intraclass coefficients (ICCs).

**Variable**	***M***	***SD***	**ICC**	**2**	**3**	**4**	**5**	**6**	**7**	**8**	**9**	**10**
Person-level												
Marital status			—	0.33^***^	−0.09	−0.09	−0.14^*^	−0.14^*^	−0.02	0.05	−0.04	−0.04
Day-Level												
Daily care			0.66	—	0.03	−0.18^**^	−0.25^***^	−0.40^***^	−0.24^***^	0.00	0.05	0.05
Daily detachment	3.01	0.77	0.39	0.01	—	0.51^***^	0.28^***^	0.38^***^	0.41^***^	0.26^***^	−0.33^***^	−0.33^***^
Daily relaxation	2.68	0.66	0.29	−0.16^***^	0.43^***^	—	0.53^***^	0.58^***^	0.81^***^	0.38^***^	−0.26^***^	−0.27^***^
Daily mastery	1.87	0.73	0.47	−0.20^***^	0.24^***^	0.43^***^	—	0.44^***^	0.55^***^	0.49^***^	−0.02	−0.01
Daily control	3.52	0.83	0.45	−0.32^***^	0.33^***^	0.56^***^	0.34^***^	—	0.66^***^	0.27^***^	−0.27^***^	−0.25^***^
Daily pleasure	3.03	0.70	0.36	−0.20^***^	0.38^***^	0.74^***^	0.47^***^	0.63^***^	—	0.44^***^	−0.34^***^	−0.31^***^
Daily relatedness	2.52	0.66	0.30	0.02	0.21^***^	0.33^***^	0.38^***^	0.23^***^	0.51^***^	—	−0.14^*^	−0.12
Daily stress	1.99	0.82	0.33	0.04	−0.29^***^	−0.21^***^	−0.04	−0.23^***^	−0.29^***^	−0.13^***^	—	0.93^***^
Daily worry	2.18	0.82	0.67	0.05	−0.30^***^	−0.22^***^	−0.05^*^	−0.21^***^	−0.27^***^	−0.11^***^	0.90^***^	—

*Correlations above the diagonal refer to person-level data (Level 2) with day-level measures aggregated at the person-level and are based on the mean of each day (4 periods). Correlations below the diagonal are derived from Level 1 data and are based on the mean of each day (4 periods). Marital status = single (0), in a relationship (1). Daily care = care for a dependent. Recovery experiences, stress and worry are adapted to the pandemic context. Daily relatedness = daily social relatedness*.

* p <0.05;

** p <0.01;

*** *p <0.001*.

### Analyses

#### Response Lag Effect

Table 2 present the results of the multilevel structural equation model predicting pandemic-related momentary negative mood. Results show that there is a positive relationship between the previous period's mood (*t–*1) and mood at a given period (*t*). Hypotheses 1a and b are thus supported.

**Table 2:** Results of the multilevel structural equation model estimating relationships between pandemic-related momentary negative mood and recovery experiences.

	**Predicting stress**						**Predicting worry**					
	**Period 2**		**Period 3**		**Period 4**		**Period 2**		**Period 3**		**Period 4**	
**Predictor**	**Est**.	***SE***	**Est**.	***SE***	**Est**.	***SE***	**Est**.	***SE***	**Est**.	***SE***	**Est**.	***SE***
**Level 1**												
Stress (*t–*1)	0.26^***^	0.04	0.24^***^	0.04	0.23^***^	0.05	–		–		–	
Worry (*t–*1)	–		–		–		0.21^***^	0.03	0.25^***^	0.05	0.18^**^	0.05
Detachment	−0.15^***^	0.02	−0.15^***^	0.03	−0.22^***^	0.03	−0.14^***^	0.02	−0.13^***^	0.03	−0.23^***^	0.03
Relaxation	−0.09^**^	0.03	−0.07^*^	0.04	−0.12^**^	0.04	−0.09^***^	0.02	−0.08^**^	0.03	−0.14^***^	0.03
Mastery	−0.10^**^	0.03	−0.05^*^	0.03	−0.05	0.03	−0.10^***^	0.03	−0.02	0.02	−0.08^*^	0.03
Control	−0.09^***^	0.02	−0.09^**^	0.03	−0.10^**^	0.03	−0.07^***^	0.02	−0.07^**^	0.02	−0.08^*^	0.03
Pleasure	−0.13^***^	0.02	−0.10^**^	0.03	−0.12^***^	0.03	−0.12^***^	0.02	−0.09^***^	0.02	−0.09^**^	0.03
Relatedness	−0.09^**^	0.03	−0.07^*^	0.04	−0.12^**^	0.04	−0.09^***^	0.02	−0.08^**^	0.03	−0.14^***^	0.03
**Interactions – Experiences**												
Detach. * Mood (*t–*1)	0.01	0.02	−0.05	0.04	−0.03	0.02	0.03	0.02	−0.03	0.04	−0.04^**^	0.02
Relax. * Mood (*t–*1)	−0.02	0.03	−0.08	0.05	−0.03	0.08	0.02	0.02	−0.04	0.03	−0.05	0.03
Mastery * Mood (*t–*1)	−0.02	0.03	−0.07^*^	0.04	−0.02	0.03	0.02	0.02	0.00	0.03	−0.00	0.03
Control * Mood (*t–*1)	−0.05^*^	0.02	−0.03	0.03	−0.04	0.04	−0.03	0.02	−0.03	0.04	−0.10^*^	0.04
Pleasure * Mood (*t–*1)	−0.03	0.02	−0.06^*^	0.03	−0.03	0.02	0.02	0.02	−0.03	0.03	−0.06	0.03
Related. * Mood (*t–*1)	−0.00	0.03	−0.04	0.03	−0.05^*^	0.02	−0.00	0.04	−0.04	0.04	−0.02	0.02

*n = 264. t – 1 = we predicted mood at a given period (t) from the previous period's mood (t – 1). Detach. = detachment. Relax. = relaxation. Related. = relatedness*.

* p <0.05;

** p <0.01;

*** *p <0.001*.

#### Recovery Experiences

Table 2 also presents standardized beta coefficients and standard errors for each interaction between recovery experiences and previous period–s mood (*t*−1) on mood at a given period (*t*). Hypothesis 2 stated that recovery experiences will moderate the within-person lag effect of pandemic-related stress between time periods. Psychological detachment and relaxation did not moderate the within-person lag effect of pandemic-related stress between time periods (independently of the period of the day), thus not supporting hypotheses 2a and 2b, respectively. Hypothesis 2c was partially supported. The positive relation between pandemic-related stress at time periods 2 and 3 significantly decreased with increasing levels of mastery. Analysis of standardized simple slopes showed that, at low levels of mastery, stress at period 2 was positively related to stress at period 3 (−1 SD; standardized simple slope = 0.36, *p* < 0.001) and this relationship was weaker at high levels of mastery (+1 SD; standardized simple slope = 0.22, *p* < 0.001). Hypothesis 2d is also partly supported. More precisely, the positive relation between pandemic-related stress at time periods 1 and 2 was weaker when control was high (+1 SD; standardized simple slope = 0.21, *p* < 0.001) compared to when it was low (−1 SD; standardized simple slope = 0.33, *p* < 0.001). As well, the results partially supported hypothesis 2e. The positive relationship between pandemic-related stress at time periods 2 and 3 was weaker when participants reported high levels of pleasure (+1 SD; standardized simple slope = 0.23, *p* = 0.003) compared to when they expressed lower levels (−1 SD; standardized simple slope = 0.35, *p* < 0.001). Finally, relatedness also moderated the within-person lag effect of pandemic-related stress. The positive relationship between stress at time periods 3 and 4 was weaker when participants reported higher levels (+1 SD; standardized simple slope = 0.25, *p* = 0.052) of relatedness compared to when they expressed lower levels (−1 SD; standardized simple slope = 0.36, *p* < 0.001).

Hypothesis 3 concerned pandemic-related worry and stated that recovery experiences will moderate the within-person lag effect of this mood between time periods. Psychological detachment moderated the within-person lag effect of pandemic-related worry. The positive relationship between worry at periods 3 and 4 was weaker when participants reported higher levels (+1 SD; standardized simple slope = 0.17, *p* = 0.003) of detachment compared to when they expressed lower levels (−1 SD; standardized simple slope = 0.27, *p* < 0.001). Relaxation, mastery, pleasure and relatedness did not moderate the within-person lag effect of pandemic-related worry between any time periods, giving no support to Hypotheses 3b, 3c, 3e, and 3f, respectively. Hypothesis 3d was partly supported. The positive relation between worry at periods 3 and 4 was weaker when control was high (+1 SD; standardized simple slope = 0.18, *p* = 0.046) compared to when it was low (−1 SD; standardized simple slope = 0.36, *p* = 0.003).

#### Contextual Variables

This section presents the effects of status and/or having dependents on the relationship between recovery experiences and pandemic-related momentary negative mood. Results are presented in Table 3.

**Table 3:** Results of the multilevel structural equation model estimating relationships between pandemic-related momentary negative mood, care for a dependent, marital status, and recovery experiences.

	**Predicting stress**						**Predicting worry**					
	**Period 2**		**Period 3**		**Period 4**		**Period 2**		**Period 3**		**Period 4**	
**Predictor**	**Est**.	***SE***	**Est**.	***SE***	**Est**.	***SE***	**Est**.	***SE***	**Est**.	***SE***	**Est**.	***SE***
**Interactions - Care (Level 1)**												
Detach. ^*^ Care ^*^ Mood (*t*−1)	0.00	0.05	0.08	0.05	0.10^*^	0.04	0.02	0.04	0.04	0.04	0.10	0.05
Relax. ^*^ Care ^*^ Mood (*t*−1)	0.09	0.07	0.03	0.04	0.12	0.14	0.04	0.07	0.11^*^	0.05	0.12	0.07
Mastery ^*^ Care ^*^ Mood (*t*−1)	0.06	0.08	0.05	0.05	0.01	0.06	0.07	0.07	0.03	0.06	0.05	0.07
Control ^*^ Care ^*^ Mood (*t*−1)	0.03	0.04	0.06	0.05	0.06	0.07	0.04	0.04	0.11^*^	0.05	0.10	0.07
Pleasure ^*^ Care ^*^ Mood (*t*−1)	0.10	0.06	0.07	0.05	−0.00	0.05	0.05	0.05	0.10^*^	0.04	0.03	0.06
Related. ^*^ Care ^*^ Mood (*t*−1)	0.07	0.07	0.06	0.04	−0.02	0.04	−0.01	0.05	0.06	0.04	−0.03	0.05
**Interactions - Status (Level 2)**												
Detach. ^*^ Status ^*^ Mood (*t*−1)	0.01	0.05	−0.08	0.05	−0.04	0.04	−0.06	0.04	0.01	0.06	−0.02	0.04
Relax. ^*^ Status ^*^ Mood (*t*−1)	−0.03	0.05	0.02	0.08	−0.03	0.21	−0.04	0.04	−0.01	0.05	−0.07	0.06
Mastery ^*^ Status ^*^ Mood (*t*−1)	0.00	0.06	−0.02	0.09	−0.07	0.05	−0.03	0.05	−0.05	0.06	−0.09	0.05
Control ^*^ Status ^*^ Mood (*t*−1)	0.09^*^	0.04	0.03	0.06	0.01	0.07	0.00	0.04	0.03	0.05	−0.01	0.08
Pleasure ^*^ Status ^*^ Mood (*t*−1)	−0.04	0.05	0.02	0.07	−0.01	0.05	−0.05	0.05	0.03	0.04	−0.05	0.06
Related. ^*^ Status ^*^ Mood (*t*−1)	−0.00	0.05	0.03	0.05	0.02	0.05	−0.04	0.04	−0.00	0.06	0.07	0.05
**Interactions with Care (Level 1) and Status (level 2)**												
Detach.*Care*Status*Mood (*t*−1)	0.05	0.11	−0.16	0.11	−0.27^**^	0.09	0.01	0.08	0.03	0.07	−0.18	0.12
Relax.*Care*Status*Mood (*t*−1)	0.06	0.20	−0.09	0.20	−0.36	0.33	0.00	0.10	−0.11	0.11	−0.10	0.15
Mastery*Care*Status*Mood (*t*−1)	−0.13	0.19	−0.16	0.10	−0.18	0.11	−0.33^*^	0.15	−0.01	0.10	−0.14	0.12
Control*Care*Status*Mood (*t–*1)	0.06	0.09	−0.19	0.10	−0.34^*^	0.13	−0.02	0.07	−0.13	0.09	−0.21	0.14
Pleasure*Care*Status*Mood (*t*−1)	0.10	0.12	−0.03	0.09	−0.35^***^	0.09	0.05	0.11	−0.04	0.07	−0.13	0.13
Related.*Care*Status*Mood (*t*−1)	0.10	0.16	−0.18^*^	0.09	−0.11	0.08	0.14	0.10	−0.19^*^	0.09	−0.12	0.11

n = 264; Period t – 1 = we predicted mood at a given period (t) from the previous period's mood (t – 1). Care = care for a dependent; Status = marital status;

* p <0.05;

** p <0.01;

*** *p <0.001*.

##### Psychological Detachment

**Figure 2:**
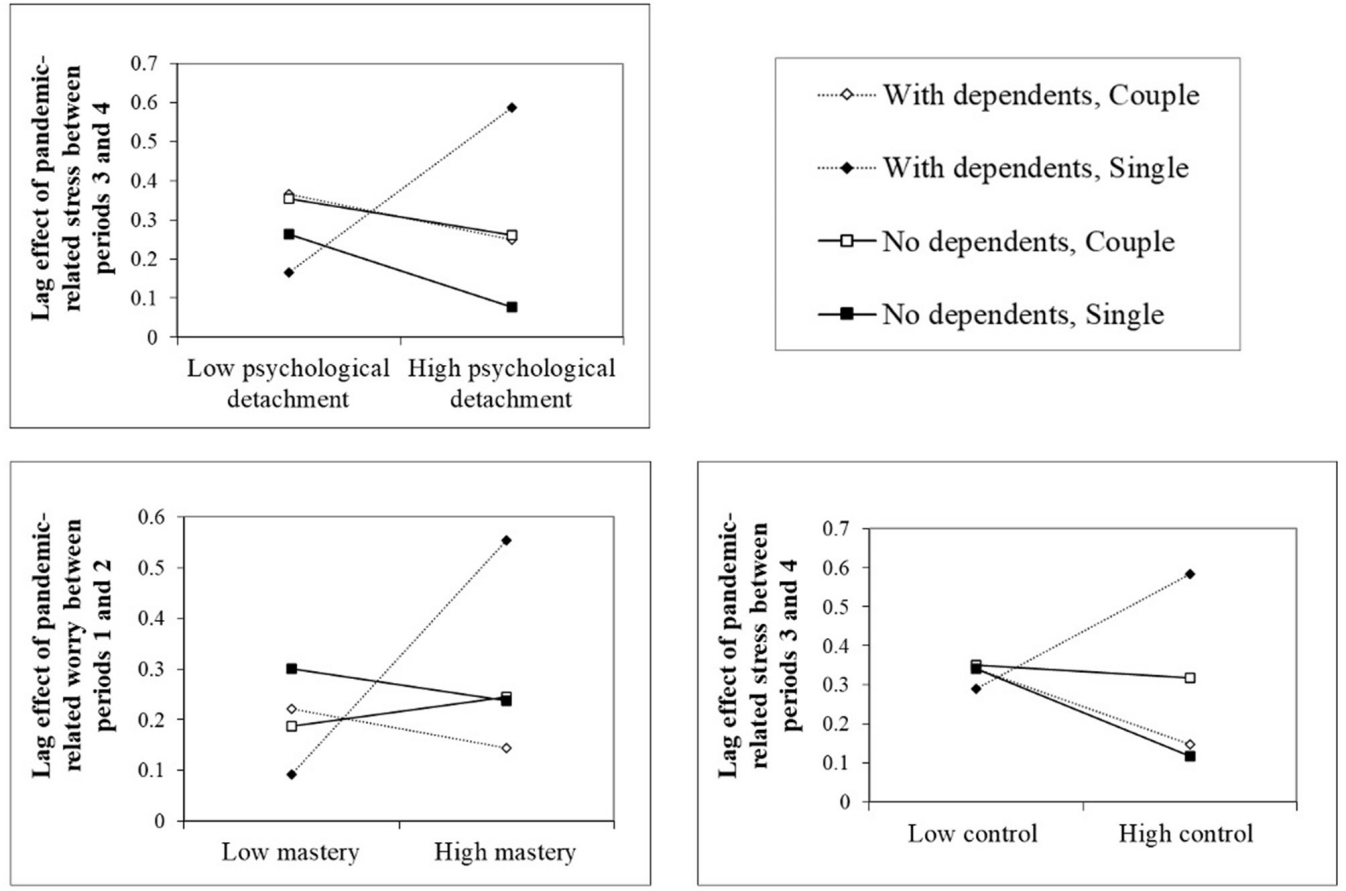
The 4-ways interactions between recovery experiences, care for dependent, status and mood.

There was a significant 4-way interaction between psychological detachment, care for dependent, marital status and mood [i.e., Detach.*Care*Status*Mood (*t*−1)] predicting the lag effect of pandemic-related stress between periods 3 and 4 (see Figure 2). Psychological detachment significantly moderated the within-person lag effect of stress among singles (with and without dependents) while it had no effect among individuals in a couple relationship. Analysis of standardized simple slopes showed that the more singles with dependents reported high levels of psychological detachment, the stronger the pandemic-related stress lag effect between time periods 3 and 4 *(b* = 0.18; *p* = 0.005). Thus, for these individuals, a low level of detachment prevented persistence of stress between the two periods (−1 SD; standardized simple slope = 0.16, *p* = 0.303), whereas a high level of detachment (+1 SD; standardized simple slope = 0.58, *p* < 0.001) did the opposite. Conversely, the more singles with no dependents experienced detachment, the less stress persisted between periods 3 and 4 (*b* = –0.08; *p* = 0.044); the betas of standardized simple slopes dropped from 0.27 (*p* = 0.057) for a low level of detachment to 0.08 (*p* = 0.546) for a high level. For worry, neither status nor having dependents or not influenced the effect of recovery experiences on the within-person lag effect.

##### Relaxation

There was no significant 4-way interaction between relaxation, care for dependent, marital status, and mood [i.e., Relax.*Care*Status*Mood (*t*-1)] predicting the lag effect of pandemic-related stress and worry. However, the 3-way interaction between relaxation, care for dependent and mood [i.e., Relax.*Care*Mood (*t*-1)] predicting the lag effect of worry between time periods 2 and 3 was significant. Standardized simple slopes analysis showed that the more individuals without dependents reported relaxation, the weaker the lag effect of worry between periods 2 and 3 (*b* = −0.07, *p* = 0.052; marginally significant), although it remains significant for each level of relaxation (standardized simple slope at −1 SD = 0.41, *p* < 0.001; standardized simple slope at +1 SD = 0.25, *p* < 0.001). However, for individuals with dependents, relaxation did not moderate the within-person lag effect of pandemic-related worry between those same time periods (*b* = 0.04, *p* = 0.310).

##### Mastery

There was no significant 4-way interaction between mastery, care for dependent, marital status, and mood [i.e., Mastery*Care*Status*Mood (*t*−1)] predicting the lag effect of pandemic-related stress. However, there was a significant 4-way interaction between mastery, care for dependent, status, and mood [i.e., Mastery*Care*Status*Mood (*t*−1)] predicting pandemic-related worry between time periods 1 and 2 (see Figure 2). Mastery had a significantly different effect (i.e., an inverse effect) on the persistence of worry between periods 1 and 2 among singles with and without dependents (*b* = 0.27, *p* = 0.046), but did not significantly alter the persistence of worry for anyone (*b* = −0.03, *p* = 0.361 for singles with no dependents and *b* = 0*.2*3*, p* = 0.073 for those with dependents).

##### Control

There was a significant 4-way interaction between control, care for dependent, status and mood [i.e., Control*Care*Status*Mood (t−1)] predicting the lag effect of pandemic-related stress between periods 3 and 4 (see Figure 2). Control moderated the persistence of stress between periods 3 and 4 among singles with no dependents (*b* = 0.11, *p* = 0.051; marginally significant), that is, it inhibited stress when the person reported a high level of control (+1 SD; standardized simple slope = 0.12, *p* = 0.343), whereas it remained significant when low levels were reported (−1 SD; standardized simple slope = 0.34, *p* = 0.007). The 3-way interaction between control, status, and mood [i.e., Control*Status*Mood (*t*−1)] predicting the lag effect of stress between time periods 1 and 2 was also significant. Standardized simple slopes analysis showed that the more singles reported control, the weaker the lag effect of stress between periods 1 and 2 (*b* = −0.10, *p* = 0.004), although it remains significant for each level of control (standardized simple slope at −1 SD = 0.43, *p* < 0.001; standardized simple slope at +1 SD = 0.18, *p* = 0.022). Finally, there was a significant 3-way interaction between control, care, and mood [i.e., Control*Care*Mood (*t*−1)]. Control had a significantly different effect (i.e., an inverse effect) on the persistence of worry between periods 2 and 3 for those with dependents compared to those who had none (*b* = 0.11, *p* = 0.011), whether they were in a couple or not, but did not significantly alter the persistence of worry for anyone (*b* = −0.06, *p* = 0.145 for those with no dependents and *b* = 0.06*, p* = 0.279 for those with dependents).

##### Pleasure

There was a significant 4-way interaction between pleasure, care for dependent, status and mood [i.e., Pleasure*Care*Status*Mood (t−1)] predicting the lag effect of pandemic-related stress between periods 3 and 4 (see Figure 2). Singles with dependents who reported feeling high levels of pleasure also reported a stronger persistence of pandemic-related stress between periods 3 and 4 (*b* = 0.13, *p* = 0.007), and that persistence remained significant regardless of the level of pleasure (standardized simple slope at SD −1 = 0.28, *p* = 0.034; standardized simple slope at SD +1 = 0.54, *p* < 0.001). Conversely, the more persons in a couple relationship with dependents reported having feelings of pleasure between periods 3 and 4, the less the lag effect of stress (*b* = 0.14, *p* = 0.026), and that lag effect was not significant when the level of pleasure was high (+1 SD; standardized simple slope = 0.12, *p* = 0.304), whereas it persisted for lower levels of pleasure (−1 SD; standardized simple slope = 0.39, *p* < 0.001). There was also a significant 3-way interaction between pleasure, care for dependent and mood [i.e., Pleasure*Care *Mood (t−1)] predicting the lag effect of pandemic-related worry between periods 2 and 3. The more singles with no dependents reported having feelings of pleasure, the less worry persisted, although it remained present (standardized simple slope at SD −1 = 0.40, *p* < 0.001; standardized simple slope at SD +1 = 0.27, *p* < 0.001).

##### Relatedness

There was a significant 4-way interaction between relatedness, care for dependent, status, and mood [i.e., Relatedness*Care*Status*Mood (*t*−1)] predicting the lag effect of pandemic-related stress between periods 2 and 3 (see Figure 2). Relatedness attenuated the lag effect of stress between periods 2 and 3 among singles with no dependents (*b* = −0.10, *p* = 0.015) although it remained significant (standardized simple slope at SD −1 = 0.46, *p* < 0.001; standardized simple slope at SD +1 = 0.26, *p* < 0.001). Relatedness also moderated the lag effect of worry between periods 2 and 3 among single (see Figure 2). More precisely, relatedness had a significantly different effect (i.e., an inverse effect) on the persistence of worry between periods 2 and 3 among singles with and without dependents (*b* = 0.17, *p* < 0.001), but did not significantly alter the persistence of worry for anyone (*b* = −0.08, *p* = 0.169 for those with no dependents at *b* = 0.09*, p* = 0.175 for those with dependents).

## Discussion

The primary aim of this study was to determine if there is a moment-to-moment response lag effect of individuals' experience of pandemic-related negative mood during the same day among those who are in voluntary self-isolation. Since, at the time and in many places, periods of isolation or at least various physical distancing measures were expected to continue to be in effect until the pandemic could be controlled, it was important to determine, first of all, if stress and worry tended to persist from one moment to another. To achieve this, we asked 264 participants in voluntary self-isolation to complete 4 diary entries per day on 7 consecutive days in which they indicated their momentary pandemic-related negative mood as well as their recovery experiences during the last time period. The findings show that there is a lagged effect of both pandemic-related negative mood from one period of the day to another during the same week among persons in self-isolation, thus supporting Hypotheses 1a and b. Concretely, this means that when a person is stressed or worried at a moment during the day, it is likely that they will continue to be in this state during the hours to follow and throughout the rest of the day. Those results are in line with studies about emotional inertia, indicating that negative mood is predicted by previous negative mood in the preceding hours (Suls et al., 1998; Kuppens et al., 2010).

Subsequently, knowing that such contamination exists, it was essential to find ways to prevent or minimize stress and worry persistence from one moment to another in order to avoid these states from becoming chronic. Indeed, this state of negative emotional inertia, or a lack of variability in affective mood, have been associated with psychological maladjustment and depression (Kuppens et al., 2010, 2012). The secondary aim was thus to identify strategies that would make it possible to attenuate such a lag effect and thereby contribute to preserving the mental health of persons who are self-isolating. The results of our study suggest that certain recovery experiences could influence this spillover. In fact, all of the recovery experiences measured did influence the response lag effect of pandemic-related negative mood at some moment during the course of the day, but careful examination of our results in line with the third aim of this study reveals that these experiences are effective at certain moments of the day and not at others and that some of these experiences can even be detrimental for some persons at the same time as they are particularly beneficial for others. The results obtained are presented in detail and discussed in terms of the domestic burden experienced during and as a consequence of the pandemic, and considering the person's family environment (i.e., in a couple relationship or not and with dependents or not).

First, taking control shows promising potential as a recovery strategy, since it generally allowed respondents to lower the lag effect of stress in the morning and worry in the evening. Thus, starting and finishing the day by taking control of one's schedule, rather than being controlled by it, fosters a decrease in subsequent pandemic-related negative mood. Special attention should thus be given to taking the time to set up one's schedule at the beginning of the day, in order to feel less under pressure later, and also to deciding how one will spend the evening, in order to feel less worry at bedtime. These results concur with those of other studies showing that managing one's time is a good way to mitigate negative mood (Häfner et al., 2014; Grissom et al., 2015; Aeon and Aguinis, 2017), and that control experiences facilitate recovery from demands (Bennett et al., 2018). Furthermore, control is particularly effective for lowering persistent stress in the evening for those who are singles without dependents, but tends to have the inverse effect on worry, depending on whether the person has dependent(s) or not [rather positive for those without dependent(s) and somewhat negative for those with dependent(s)]. These results are not surprising, since, when one has no dependents (and especially when one has no partner), one decides how to spend time without consultation or compromise and no potential negative impact on anyone, which maximizes the chances that this strategy will be beneficial for the person exercising control. For those who live with others, choices can have consequences (sometimes positive, sometimes negative), and it can be more difficult to have a sense of complete control over one's schedule and thus to benefit from this type of recovery experience. This also explains the significantly inverse effect observed between persons with dependents and those without in regard to feelings of worry after having decided how to spend one's time in the afternoon.

In addition to control, detachment also makes it possible to minimize pandemic-related negative mood, but only at the end of the day (lag effect between time periods 3 and 4). In fact, it generally allowed respondents to minimize the lag effect of worry, and for singles with no dependents, of stress as well. These results concur with those of several studies that have highlighted that detachment is particularly effective for recovering from strain, such as, for example, after a day of work [see Sonnentag and Fritz (2015) for a review]. One of the reasons detachment from the pandemic may be so effective in the evenings is that, during the day, people probably switch to doing something else (rather than thinking about the pandemic; e.g., working from home), rather than deliberately seeking to detach. Detachment may well be contextual rather than intentional such that it has no recovery function in this particular context. The person is not thinking about the pandemic simply because they are otherwise occupied. This would be why there is no influence on pandemic-related negative mood.

Relaxation has no impact on the lag effect of pandemic-related negative mood at any time of the day, except for those who have no dependents, for whom the lag effect of worry diminishes as relaxation increases at the end of the day. This result may seem surprising, since engaging in activities that bring out positive emotions with little investment of energy, such as taking a nap, are commonly believed to improve mood (Daiss et al., 1986; Kaida et al., 2007). In principle, relaxation can be restful and generate positive emotions in those who relax, thus helping them to recover (Bennett et al., 2018). However, Lazarus and Mayne (1990) stress that sometimes relaxation can also have negative effects, if the techniques are not adapted to the individual or their particular situation. In this regard, the results of the present study suggest that persons with no dependents may be more disposed to benefit from relaxation when self-isolating than those with dependents. In fact, when the latter manage to have a moment to relax, they may be less inclined to take advantage of it and quite likely to see any beneficial effects evaporate as soon as they are back in the middle of the hectic day typical for those with full-time dependents and more limited resources due to schools, daycare and homecare being shut down. The design of the present study does not enable measurement of the effect of relaxation while it is being experienced, only its subsequent effect, in the hours that follow. We recommend that future studies use a concomitant measure (e.g., experience sampling) of recovery experiences and mood in order to determine whether an immediate effect can nonetheless be felt by persons with dependents. It would also be relevant to investigate specific relaxation techniques rather than the experience felt, in order to determine whether some of these could be more beneficial than others as a function of the individual's life situation.

Relatedness proved to be particularly effective at the end of the day, since it generally enabled participants to lower the lag effect of stress in the evening, and for singles with no dependents it did so at midday only. Among singles only, it also decreased the lag-effect of worry at midday. Connecting and feeling close to others in a context of social distancing thus seems important for reducing the lag effect of pandemic-related negative mood. It is not surprising that relatedness stands out as a particularly effective strategy at midday among singles in the context of self-isolating. In fact, experiencing connection and intimacy is probably very significant and of great value when one does not share daily life with a partner. Thus, having the chance to experience relatedness is all the more restorative, given the rarity of this resource (Hobfoll, 1989) in the context of a pandemic such as the current one, when one is isolated at home and separated from loved ones. Given the social distancing measures in place, these recovery experiences probably occurred virtually in a number of cases. Further research would be required to determine whether virtual experiences of relatedness are as effective as in-person ones for reducing the stress and worry lag-effect from one moment to another during the day.

The results also reveal that mastery at midday (between time periods 2 and 3) generally diminishes the lag effect of participants' stress, and diminishes worry in the morning among singles. Finding a moment to broaden one's horizons in the afternoon, through new experiences such as making bread or learning more about a topic of interest (other than COVID-19), may help temper the level of stress experienced. These results concur with those of other studies showing that experiences of mastery (Bennett et al., 2018) can be restorative in daily life, since they make it possible to compensate for expended resources by “filling up” with new experiences. Broadening one's horizons may make it possible to enrich oneself by exploring new avenues when outside resources are temporarily unavailable due to more limited contacts with others because of self-distancing measures. This may explain why this strategy was particularly effective among singles for whom resources from the outside (i.e., a partner) are even more limited in context of a pandemic.

In the vein of regenerating resources, our data show that feeling pleasure also generally enables participants to diminish the lag effect of pandemic-related stress at midday. Furthermore, when these feelings are strong, they even make it possible to prevent the lag effect of stress among those in a couple relationship with dependents in the evenings. Feeling pleasure allows the person to replenish their resources through the strong positive emotions it generates (van Hooff and de Pater, 2017) and fosters recovery in daily life (Bennett et al., 2018). Numerous studies have shown that pleasure makes it possible to manage stress and to recover (Esch and Stefano, 2004; Oerlemans et al., 2014; van Hooff and de Pater, 2017), and our study adds to previous findings by highlighting that in a context of self-isolation, feeling pleasure is lifesaving if the person takes responsibility for the potential consequences. In fact, we posit that feeling pleasure during self-isolation is a deliberate action, that is, one chooses to prioritize pleasure over other tasks one could engage in. Thus, for those who have no dependents or who are in a relationship and have dependents, feeling pleasure is especially restorative, since the time devoted to pleasure will not have to be compensated for later on (if one has dependents, one's partner is there to take care of at least some of their needs). In the case of persons in a relationship who have dependents, choosing to engage in pleasurable experiences is potentially beneficial to all concerned [i.e., crossover of the effect of recovery experiences among couples (Park and Fritz, 2015)]. Furthermore, there is no great consequence for dependents even though they do not share in the moments of pleasure, since the partner is there to look after them. On the other hand, we posit that singles with dependents may feel guilty for deliberately choosing to do something that gives one pleasure in the evening (rather than taking care of dependents), which could explain the deleterious effect observed. Feelings of pleasure imply having made a choice that has the potential to cause moral harm to the dependent, which could be what leads to feelings of guilt (Tangney, 1990) because it is an experience fundamentally oriented toward the self rather than another. In focusing on oneself rather than the dependent or one's responsibilities in relation to them, guilt feelings can easily arise. When one has dependents, in the context of self-isolation, one is expected to be their teacher too because schools are closed and/or the dependents are likely to be among the high-risk populations for the virus, so the demands are enormous and pressure is at its peak. This is especially true for singles, since they have little room to engage in compensatory activities because no one can take over. In this context, it is highly probable that one would constantly have the impression that one is not doing enough, since one's domestic burden is much heavier than normal and carried alone. When singles with dependents feel pleasure, it is possible that guilt catches up to them (and potentially, the domestic burden of the responsibilities they have not dealt with during those much longed-for moments). They regret having let go and fulfilled their need for pleasure and broadening their horizons, rather than focusing exclusively on their duty/responsibilities, and guilt is the price they pay for it. This is a very disturbing finding, since these persons also need to replenish their resources and recover. It is all the more worrisome because their domestic burden is probably greater than that of persons with a partner or without dependents.

Like pleasure, detachment from the pandemic (i.e., not thinking about it) during the evening can also have a deleterious effect on singles with dependents, by worsening the lag effect of stress (even though it diminishes it among singles with no dependents). One might think that distancing oneself from the pandemic could be a good strategy for diminishing the negative mood of singles with dependents during such uncertain times. After all, distancing oneself from a source of strain has been shown to be effective in diminishing negative mood (Sonnentag and Bayer, 2005) and stress in the context of work (Sonnentag and Fritz, 2015), as well as for recovering between periods of work (Wendsche and Lohmann-Haislah, 2017). Furthermore, our results show that in the evening, detachment made it possible for stress levels to fall in singles with no dependents, and for worry to decrease generally. In most cases, this is thus a good way to regulate negative mood at the end of the day. However, we posit that when one is absorbed in one's daily tasks (i.e., overloaded) detachment is not a consequence of an intentional act, in which one wishes to distance oneself from the source of stress (i.e., the pandemic), as is the case when one wants to take a step back from work in the evening or on the weekend [e.g., going for a run or playing with one's children so as not to think about work, the context in which detachment has previously been studied (Sonnentag and Fritz, 2015)]. Thus, one does not detach voluntarily when one is single with dependents in the context of a pandemic. One does so simply because one has no time to think about the pandemic, given all the other things one must think about. In other words, when singles with dependent report being highly detached they do so because of being overworked and overloaded, which results in still being tense (i.e., stressed) at bedtime. Consequently, we believe that the relationship observed is as follows: the more singles with dependents are overloaded at the end of the day, the less they think about the pandemic, and the more they are detached, but also, the more their stress has increased, peaking at bedtime. Thus, it is probable that the magnitude of their stress in relation to the pandemic catches up with them or hits them all the more because they have just not been able to reflect at all in the evening hours, during which they experienced the full weight of their domestic burden (demands) due to the absence of resources available to them to respond to these demands, which leaves them facing the situation alone. This explanation is supported by the fact that detachment influences the lag effect of stress, but not that of worry. Detachment thus has a harmful effect only at the end of the day and only among those who are extremely overloaded and have no support from a partner to share this domestic burden (i.e., singles with dependents), whereas it has a beneficial effect for those with a lesser load (i.e., singles with no dependents), for whom detachment from the pandemic is probably intentional and genuinely represents choosing not to think about the pandemic.

### Practical Implications

We recommend that mental health experts inform their clients about the importance of engaging in activities that allow them to recover resources expended during the day, while being aware that time is a limited resource that must be invested in a way that balances responding to requests and recovering resources. Everything is a question of balance, and when a person invests time in activities that allow them, for example, to feel pleasure, the weight of tasks that must be accomplished continues to accumulate. This is why singles with dependents may be more at risk of being in a negative mood and experiencing repercussion from taking time for themselves. The fact that their resourcing experiences sometimes generate an increase of negative mood spillover from one measurement time to the next is very concerning and suggests that accumulating this lagged effect could be particularly deleterious for them over time, putting them at risk of developing mental health issues. In fact, according to COR (Hobfoll, 1989) a “loss spiral” can set in: affected individuals can lack resources (e.g., support from a partner) to compensate for those they have expended, which can accentuate the acute stress and worry they feel when they have to respond to their usual demands, such as continuing to care for dependents in daily life (Sonnentag and Zijlstra, 2006). The results observed with singles with dependents reflect this tendency and lead us to emphasize how important it is for this group and their family members to be vigilant about their well-being. We strongly encourage singles with dependents who are cut off from resources due to distancing measures to seek out ways to lighten their load and obtain support as soon as possible. It is imperative that they have resources available to them that can provide support that is both instrumental (someone who can take over and give them a break, concrete assistance) and emotional (a connection to lessen solitude).

Regarding the guilt that may accompany feelings of pleasure, one strategy to reduce guilt that could be helpful draws upon work-family enrichment theory (Greenhaus and Powell, 2006). This approach suggests that individuals would benefit from shifting their perspective and trying to view their work as an investment that can be beneficial and enriching for their role in supporting a dependent (i.e., parent or caregiver). To do so, they could apply a strategy of cognitive reformulation, recognized as effective for quieting the cognitive dissonance (Festinger, 1957) experienced when parents engage in behaviors (e.g., doing something to take care of themselves) that are in conflict with their personal values [i.e., degree of importance ascribed to the sphere of family life (Johnston and Swanson, 2007)]. Singles with dependents could in fact adopt a perspective of work-family enrichment (Greenhaus and Powell, 2006), and acknowledge that their investment in leisure activities for themselves (i.e., feeling pleasure in the evening) enriches their emotional competence, for example, which is beneficial to their well-being and role as a parent or caregiver. By acknowledging that leisure generates resources and that it can be beneficial to invest one's resources in that sphere of life, they could reduce the guilt they associate with pleasure in the evening. Put simply, one has to put on one's own oxygen mask before putting one on someone else. In this vein, exercising self-compassion is also a good way to self-care, since this has been shown to be effective when one is particularly vulnerable or stressed[(see Neff and Germer (2013) for a guide to self-compassion].

In addition to recommendations for individuals in this situation, we strongly encourage communities to put in place measures to support persons who are particularly affected by the consequences of physical distancing measures. We salute the initiatives observed throughout our communities, for example, volunteers who rock babies to sleep virtually or in a stroller or run errands for single parents. We strongly recommend not to hesitate to aid those who need it in any way we can during the pandemic (and afterwards), of course making sure to respect recommended public health measures.

### Theoretical Implications

This study significantly contributes to the literature on resources and recovery (Sonnentag et al., 2017). To the best of our knowledge, this is the first study indicating that recovery experiences constitute beneficial stress management strategies during a major crisis such as being locked down during the COVID-19 pandemic, previous studies having mostly focused on recovery experiences in the context of work or work-life balance.

Furthermore, this is the first study showing that recovery experiences are not a panacea and may be detrimental in some contexts (i.e., having a partner or not and having dependents during a pandemic). This study stressed the importance of evaluating domestic burden when measuring detachment, because these two concepts can overlap. This also highlights that psychological detachment is probably an effective strategy if there is a certain type of intentionality. Studies on that particular recovery experience should take this into account and evaluate it in order to better understand the results obtained.

### Limitations

However, this study also has some limitations. Since this study was conducted over 1 week, the long-term consequences of lockdown were not considered. Longitudinal studies have shown that being locked down represents a considerable risk for the mental health of all individuals (Codagnone et al., 2020; Niedzwiedz et al., 2021). It is therefore important to establish how and when short-term strategies to manage one's negative mood are no longer sufficient. It would also be important for future studies on the effects of physical distancing during this pandemic to evaluate the long-term effect of such measures on the mental health of singles with dependents. Although their level of stress and worry was comparable to that of persons in a couple relationship and/or without dependents, the fact that their negative mood worsened following recovery experiences suggests that their state could deteriorate rapidly, due to a cumulative effect. It would be important to identify effective means to protect them. The use of single items to measure mood and recovery experiences is another limitation worth mentioning. Indeed, such measure may be less comprehensive and reliable to assess complex phenomenon (Loo and Kells, 1998). However, single item are appropriate when studying narrow and unambiguous concepts, as well as in the presence of situational research constrain (Wanous et al., 1997; Rossiter, 2002), such as restricted time and low energy level of participants going through a pandemic. Other studies have successfully used single item to measure mood and emotional state (Zimmerman et al., 2006). Nevertheless, future studies are needed to confirm the validity and reliability of the single items used to measure recovery experiences. Finally, the generalizability of the findings is limited by the specific characteristics of the participants who were Canadians, highly educated and mostly female. Studies should replicate the results of the present study among more diverse samples.

Nevertheless, this study brings further insight into current knowledge by indicating more specifically when and for whom recovery experiences are most effective to reduce negative mood lag effect during a major stressful event, such as a lockdown.

## Conclusion

In summary, in the present study, in addition to evaluating the effect of recovery experiences on pandemic-related negative mood lag effect, examining the differential impact on subgroups (that is, having a partner or not and having dependents or not) highlighted that recovery experiences vary depending on one's life context. Thus, although our results show that recovery experiences are mostly beneficial or neutral, the inclusion of contextual variables in our analyses allowed us to determine, to our great surprise, that certain experiences reported (i.e., detachment and feelings of pleasure at the end of the day) are in fact detrimental for singles with dependents (i.e., foster persistence of stress) or at least have a tendency to play a diametrically opposite role according to whether one has dependents or not (i.e., worry at midday relative to control experienced) and that certain experiences are not beneficial for some (i.e., detachment at the end of the day attenuates persistence of stress among singles without dependents, or relaxing at midday attenuates persistence of worry for those with no dependents, relatedness at midday attenuates worry among singles, or feeling pleasure at the end of the day attenuates the lag effect of stress in persons who are in a relationship with dependents). Recovery experiences are available to all and can easily be fostered; they are relevant and accessible means to take care of oneself in daily life during a pandemic. We do, however, encourage psychologists and specialists working in mental health to show caution so as to avoid presenting them as a panacea that can be beneficial for everyone.

## Data Availability Statement

The raw data supporting the conclusions of this article will be made available by the authors, without undue reservation.

## Ethics Statement

The studies involving human participants were reviewed and approved by Universite du Quebec a Montreal Human Sciences Faculty's Institutional Review Board (approval number 4307_e_2020). The patients/participants provided their written informed consent to participate in this study.

## Author Contributions

JM, AF, HL, and SM contributed to conception and design of the study. AF organized data collection. HL organized the database and performed the statistical analysis. JM wrote the first draft of the manuscript. AF wrote sections of the manuscript. All authors contributed to manuscript revision, read, and approved the submitted version.

## Conflict of Interest

The authors declare that the research was conducted in the absence of any commercial or financial relationships that could be construed as a potential conflict of interest.
